# Multiple aspects of matrix stiffness in cancer progression

**DOI:** 10.3389/fonc.2024.1406644

**Published:** 2024-07-02

**Authors:** Alessandro Mancini, Maria Teresa Gentile, Francesca Pentimalli, Salvatore Cortellino, Michele Grieco, Antonio Giordano

**Affiliations:** ^1^ Department of Translational Medical Sciences, University of Campania “Luigi Vanvitelli”, Naples, Italy; ^2^ BioUp Sagl, Lugano, Switzerland; ^3^ Department of Environmental, Biological and Pharmaceutical Sciences and Technologies, University of Campania “Luigi Vanvitelli”, Caserta, Italy; ^4^ Department of Medicine and Surgery, LUM University “Giuseppe De Gennaro,” Casamassima, Bari, Italy; ^5^ Laboratory of Molecular Oncology, Responsible Research Hospital, Campobasso, Italy; ^6^ Scuola Superiore Meridionale (SSM), Clinical and Translational Oncology, Naples, NA, Italy; ^7^ Sbarro Health Research Organization (S.H.R.O.) Italia Foundation ETS, Candiolo, TO, Italy; ^8^ Sbarro Institute for Cancer Research and Molecular Medicine, Center for Biotechnology, College of Science and Technology, Temple University, Philadelphia, PA, United States; ^9^ Department of Medical Biotechnologies, University of Siena, Siena, Italy

**Keywords:** ECM, matrisome, drivers, addiction, desmoplasia, matrix stiffness

## Abstract

The biophysical and biomechanical properties of the extracellular matrix (ECM) are crucial in the processes of cell differentiation and proliferation. However, it is unclear to what extent tumor cells are influenced by biomechanical and biophysical changes of the surrounding microenvironment and how this response varies between different tumor forms, and over the course of tumor progression. The entire ensemble of genes encoding the ECM associated proteins is called matrisome. In cancer, the ECM evolves to become highly dysregulated, rigid, and fibrotic, serving both pro-tumorigenic and anti-tumorigenic roles. Tumor desmoplasia is characterized by a dramatic increase of α-smooth muscle actin expressing fibroblast and the deposition of hard ECM containing collagen, fibronectin, proteoglycans, and hyaluronic acid and is common in many solid tumors. In this review, we described the role of inflammation and inflammatory cytokines, in desmoplastic matrix remodeling, tumor state transition driven by microenvironment forces and the signaling pathways in mechanotransduction as potential targeted therapies, focusing on the impact of qualitative and quantitative variations of the ECM on the regulation of tumor development, hypothesizing the presence of matrisome drivers, acting alongside the cell-intrinsic oncogenic drivers, in some stages of neoplastic progression and in some tumor contexts, such as pancreatic carcinoma, breast cancer, lung cancer and mesothelioma.

## Graphical Abstract

In physiologic conditions, cell-ECM interactions occur to regulate functions as proliferation, migration and differentiation (Soft matrix). During neoplastic progression, the matrix becomes rigid and highly dysregulated, fibrotic, playing pro-tumorigenic roles that contribute to a number of tumor hallmarks as a driving force of the oncogenic process (Stiff matrix). (BioRender.com).

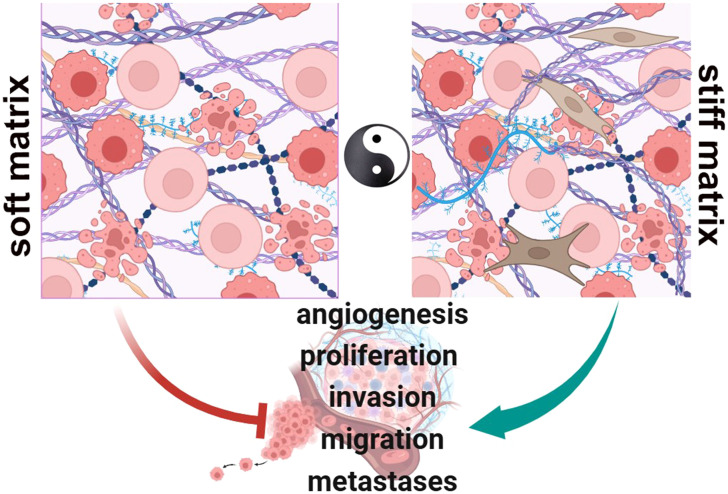

## Introduction

During development, all the tissues of multicellular organisms grow and differentiate and, the ECM plays a fundamental role regulating, at the macroscopic level, the expansion of the tissues through mechanical forces and, at the microscopic level, intervening in the processes of cell proliferation, survival, migration and others (1) The ECM is a highly complex structure present in every tissue. It is made up of a variable combination of proteins, proteoglycans and other molecules that influence a wide range of processes including cell adhesion and growth (2–4). The ECM is constantly subject to processes of remodeling, repair and renewal thanks to enzymes that cause its degradation and induce structural changes. Cell-ECM interactions regulate tissue homeostasis influencing processes such as proliferation, migration and differentiation (5). Also, the ECM mediates interaction between cells by both chemical and electrical ways (6). ECM is secreted mainly by fibroblasts, but in several specialized tissues such as cartilage or bone, it is produced by chondroblasts and osteoblasts, respectively. The physiological ECM is highly heterogeneous among organs. For example, fibroblasts are capable of synthesizing and secreting collagen I or III, elastic fibers, reticular fibers, and proteoglycans, whereas chondroblasts synthesize and secrete a cartilaginous ECM consisting of elastic fibers, collagen II, and glycosaminoglycans. Furthermore, several studies have shown that also other cells (pericytes, vascular smooth muscle cells) are able to produce ECM such as collagen IV, laminin, fibronectin (6). Given the diversity of origins of these ECM-secreting cells, their synergy contributes to the heterogeneity and complexity of the physiological ECM, which is continuously remodeled in order to adapt and maintain tissue homeostasis.

Two types of ECM have been described, the interstitial ECM and the basement membrane. The interstitial ECM represents the matrix between cells (7). This latter is composed of many structural proteins, such as collagen I and III, proteoglycans, such as versican, aggrecan, brevican, and decorins, and fibronectin that provide tissue support. Furthermore, it is also made up of other proteins such as nidogen-entactin, produced by epithelial cells, endothelial cells and myofibroblasts (6, 7). The collagen IV structural network maintains cell stability and is tightly cross-linked. On the other hand, the basement membrane is a layer of specialized ECM, positioned at the interface between parenchymal and connective tissues. The basement membrane is responsible for the isolation of the epithelium from the underlying stroma, and for the regulation of cellular organization and differentiation through interactions with the cell surface receptors. and consists of laminins, collagens (predominantly collagen IV), proteoglycans, calcium binding proteins such as fibulin, and various other structural or adhesive proteins. In particular, laminin networks, are non-covalent and are biologically more active than the collagen IV network (8). The two networks are connected to each other by nidogen which, together with other components, dynamically stabilizes the structure (9). The bone marrow, for example, is ─ not surprisingly ─ denser as it largely contains collagen IV, laminin and heparan sulfate proteoglycans (HSPG). Hemidesmosomes and integrins are cell-membrane receptors expressed in the bone marrow and bind to ECM proteins. Matrix proteins also connect to receptors such as sulfated glycolipids, dystroglycan, Lutheran glycoprotein (10). Furthermore, fine communication between cells is required to correctly assemble the basement membrane (9).

The entire ensemble of genes encoding components of the ECM and its associated proteins is called matrisome; the first are regarded as the **‘**core matrisome**’** (10, 11). The mammalian matrisome contains approximately 1,100 genes that can be gathered into different groups according to their final localization. In particular, we can recognize the core-matrisome molecules (~300 genes) and the matrix-associated molecules (~800 genes). Matrisomal proteins encoded by these genes are thought to be approximately 4% of the entire human proteome (10, 11). The core matrisome consists of ECM proteoglycans, glycoproteins, and collagens; whereas ECM-associated proteins include proteins that are structurally similar to ECM proteins and ECM remodeling enzymes (11).

External stimuli are mediated by the ECM and are transduced via adhesion molecules, integrins and other mechanotransducers (12), which connect, integrating into the focal adhesion complexes, the cellular cytoskeleton and the ECM for a mutual communication of forces between the cells and the microenvironment (13). Conformational changes in focal adhesion molecules, due to mechanotransduction mediated and regulated by various receptors, lead to cytoskeletal tension and downstream intracellular signaling, which in turn regulates migration, adhesion, proliferation and remodeling of the ECM (13).

In fibrosis, cancer, and many other non-tumoral pathological conditions, the composition of the ECM becomes dysregulated and ECM structure becomes rigid and more abundant (10), leading to a consequent imbalance of tissue homeostasis (14). Here, we provide an overview of the main changes occurring to the ECM which favor cancer progression and are increasingly recognized as potential targets for antitumor strategies.

## ECM remodeling in tumors

The tumor matrix is subjected to a high remodeling that leads to the rapid degradation of the normal, physiological matrix and its replacement with a **‘**tumor**’** matrix which can reinforce the aggressive cancer characteristics and induce the formation of bioactive fragments (known as matricryptins or matrikines) with pro-tumor features (15). Also, the matrix degradation can release growth factors/mitogens from the ECM reservoir and remove physical barriers (such as basement membranes), facilitating tumor proliferation, invasion and spreading (16). Here we focus on several aspects that characterize ECM remodeling during tumor progression.

### ECM stiffness and desmoplasia

The tumor stroma is composed of many different cell types including fibroblasts, endothelial cells and immune cells, as well as a desmoplastic matrix. Desmoplasia refers to the accumulation of dense fibrosis around the tumor and is characterized by an increase in alpha actin-positive fibroblasts with a deposition of tough ECM composed mainly of collagen, fibronectin, proteoglycans and hyaluronic acid (17). ECM stiffness is a feature of tumors that differentiates them from normal tissue (18). Alterations in ECM composition due to desmoplasia are characterized by an increase in the relative activity of matrix metalloproteases (MMPs) in several types of cancers (19) leading to an imbalance of tissue homeostasis. This results in a change of the biophysical characteristics of the ECM (20). Interstitial fibrillary collagen types I, III (21) and IV (14) are critical for alterations in tissue homeostasis associated with tumor desmoplasia. Increased collagen deposition and cross-linking, the latter mainly mediated by lysyl oxidases (LOX) and LOX-like (LOXLs) enzymes, induce ECM stiffening (22). ECM stiffness is associated with tumor growth and correlates with increased metastasis rate and poor clinical outcomes, due to resistance to therapy (18, 23). Desmoplasia not only promotes survival of tumor cells, cancer resistance to therapy and immune escape (24), but may also favor tumor development preceding tumor onset (25). Indeed, desmoplasia confers high mammographic density to the breast tissue, which represents a major risk factor for breast cancer development (25). Moreover, tumor desmoplastic ECM**/**stiffness is a fibrotic state characterized by increased deposition and altered matrix organization as well as post-translational modifications (PTM) of ECM proteins, such as hydroxylation, phosphorylation, N- and O-glycosylation, acetylation, ubiquitylation, sumoylation and methylation (26), which, in turn, activates mechanosensitive pathways within all cells present in the tumor microenvironment (TME). Among these, the remodeling of collagen/elastin (27), hyaluronic acid (28–30), and fibronectin (31–33), affects matrix stiffness by promoting the formation of the desmoplastic matrix, characterized by the growth of an ECM increased in collagen and a stroma with a high interstitial pressure, known, in its biophysical complex, as a desmoplastic reaction. This condition creates a microenvironment that favors both tumor growth and metastatic spread, constitutes a condition that hampers the right bioavailability of chemotherapy to the deepest part of the tumor. Furthermore, this stiffness can transfer biophysical signals from the ECM to the intracellular matrix via mechanical conduction, thereby changing the biological behavior of the cell. Tumors with desmoplastic/fibrotic stroma, such as breast, pancreatic, and lung cancer, are characterized by chronic inflammation, fibroblast expansion and related activation in the cancer associated fibroblasts (CAFs) phenotype, elevated angiogenesis and, in particular, increased levels of remodeling and often cross-linked matrix molecules [for review see (27, 34–36)]. Studies have also demonstrated that both collagen receptors and other classes of cell-membrane receptors, including Discoidin Domain Receptor family (DDR1 and DDR2), Integrin, and hyaluronic acid receptors (CD44, RHAMM, Toll-like receptors (TLRs), and Fibronectin receptors (α5β1, αvβ3, α4β1), β 1 (37), TRPV4 (38, 39), Piezo1 (40), Polycystin-1 (PC-1) (41, 42), CD44, RHAMM (43–47), LRP5-Tie2 (48), are involved in the mechanotransduction of the stiff matrix and their number is continuously updated, demonstrating the redundancy of the pathways. Increased tumor cells adhesion to the remodeled ECM can bypass many of the normal growth-suppressing pathways, promoting malignant transformation. The remodeling of the matrix presents parallels with what occurs in the formation of premetastatic niches. As matter of facts, the quiescent or dormant state observed in some extravasated metastatic cells may be due, at least in part, to several phenomena, such as the inability to remodel the ECM in a stiffness direction, the lesser activation of matrix receptors-dependent signal, and the synergy between the receptors of growth factors and matrix at the secondary colonization site (49). Thus, matrix remodeling that occurs as part of non-tumor-associated tissue fibrosis, can create microenvironments that facilitate primary and secondary colonization of tissue (50) hypothesizing an increasingly biophysical and biomechanical regulation of the neoplastic progression given by the matrix, by its remodeling and by the cross talk with the receptors of growth factors and cytokines.

### The role of post-transcriptional modifications (PTMs) of the ECM components

PTMs of ECM-associated proteins are important as they impart critical structural and functional characteristics to the proteins they target. PTM of matrix molecules alters interactions with other matrix molecules and receptors and can also change the charge of the molecule. PTMs are carried out by several families of intracellular and extracellular enzymes, prolyl hydroxylase, lysine hydroxylase, the extracellular LOX, transglutaminase, sulfatase, heparanase, cathepsin and the methzincine superfamily. Excessive post-translational modifications of the matrix give crucial contribution to the development of several tumor hallmarks as a driving forces of cancer progression (51). In particular, during or after the synthesis of collagen, proline and lysine residues are enzymatically transformed into 3- and 4-hydroxyproline and 5-hydroxylysine, creating hydrogen bonds that stabilize the collagen molecules and confer thermal stability to them. Moreover, this allows collagen fibrils formation, nascent collagens synthesis, as well as recognition by integrins and DDRs receptors, at least as nucleation sites favoring the assembly, organization of ECM components (52). In the same way, the glycosylation of collagen, fibronectins, laminins and proteoglycans (and their possible supramolecular complexes) is crucial for recognition by, for example, integrins and fundamental for adhesion, cell movement and proliferation (51). On the other hand, defective PTM is, for example, crucial in the deregulation of tumor suppressor proteins and oncogenes (53). Similarly, the post-transcriptionally modified matrisome participates in cancer progression and metastasis, in various ways: modulating stiffness within the TME; inhibiting or enabling recognition of matrix receptors; ectopically activating or inactivating functions within matrisome proteins; enabling or suppressing the production of cryptic active domains (such as matricryptins and matrikines) hidden within larger matrix proteins rendering them non-latent (26, 51). Differently from other diseases, data on genomic alterations and genetic mutations in the tumor ECM are relatively few. A study of 9075 tumor samples and 32 tumor types from The Cancer Genome Atlas (TCGA) Pan-Cancer cohort identified non-silent mutations in the coding regions of the matrisome, about 1800 of which have been related to PTMs that potentially can deregulate the normal function of the matrisome. Transcriptomic and proteomic studies of the TME have allowed to identify the molecular differences of the various cell populations, with respect to non-pathological states. These types of analyses do not yet allow to study the effect of the mechanical forces of the ECM on gene and protein expression. Only recently the development of technologies capable of analyzing the presence of mRNA *in-situ* has allowed this type of analysis giving beginning of the era of spatial-omics (54) which revolutionized ECM research (26, 51) (for review see (50, 51, 55–59).

### Dissecting matrisome complexity

Despite the undoubted impact that ECM proteins mutations have on normal and pathological cellular functions, few studies have focused on determining the extent of PTM alterations and mutations in ECM genes in tumors. Mutations and copy number alterations are frequent in matrisomal genes and their frequency is an independent prognostic factor related to the matrix and not to the altered oncogenic survival pathways (60, 61). The crucial role of the ECM in several aspects of cancer progression, including tumor heterogeneity and response to therapy, has been made possible in part by technological development such as mechanical probing, imaging, and proteomic methods, that overcame the constraint posed by the ECM itself due to the lack of experimental models. Moreover, to understand the matrisome, a computational approach was used by defining genes that encode core ECM proteins, or structural components of the ECM, including collagenous proteoglycans, glycoproteins, and matrix-associated proteins, matrix remodeling enzymes, proteins structurally or functionally related to matrix components, as well as secreted molecules (51, 52). Furthermore, tumor proteomics data revealed that qualitative and quantitative alterations of the matrisome contribute to tumor progression (50). ECM proteomics of tumor xenografts also demonstrated that, while stromal cells in continuous cross talk, in particular CAFs, tumor associated macrophages, cancer -associated adipocytes, are major contributors to ECM production of TME, the cancer cell also actively produces and secretes ECM proteins (49, 53, 54). **“**Omic**”** approaches made possible the discovery of ECM genes and proteins whose presence predict the prognostic outcome of patients.

## Inflammation in desmoplastic matrix remodeling

As early as 1986, Dvorak defined tumors as **“**wounds that do not heal**”**. This definition stems from the observation that tumor growth leads to a continuous lesion of the surrounding stromal tissue which also triggers a chronic inflammation in order to try to repair the lesion and restore tissue homeostasis (62). This reaction causes a continuous state of inflammation with the recruitment and concerted activity of inflammatory molecules, fibroblasts, and immune cells. As matter of fact, when a tumor lesion occurs, immune cells, such as leukocytes, macrophages and/or bone marrow-derived myeloid precursors, are recruited to the damaged site. These infiltrating immune cells, together with stromal cells, release high levels of transforming growth factor β (TGF β) into the TME, exerting pleiotropic effects on both tumor and normal cells adjacent to the lesion through autocrine and paracrine ways (63).

### The role of TGF β

As in normal and precancerous stages of cellular transformation, TGFβ exhibits tumor inhibitory features by suppressing tumor cell growth activating anti-proliferative and pro-apoptotic intracellular signaling pathways (64). This mechanism acts also disfavoring the proliferation and differentiation of cells of the innate and adaptive immune system, thus suppressing tumorigenic inflammation. However, in the late stages of carcinogenesis, TGFβ receptors genes acquire inactivating mutations that inhibit their signal transduction pathway (64) thus tumor cells become resistant to the negative regulatory effects of this cytokine by exploiting its pro-tumor action. Consequently, TGFβ activity changes from a tumor suppressive to metastasis promoting and induces modifications in the TME that ultimately support tumor growth (65). In fact, high levels of TGFβ in the stroma induce immuno-surveillance escape of tumor cells through the overproduction of cytokines and chemokines that contribute to increasing chronic inflammation (64, 65). Furthermore, tumor cells synthesized TGFβ acts indirectly on stromal cells by stimulating the production of growth and mitogenic factors, such as platelet-derived growth factor (PDGF), and inducing the trans-differentiation of stromal progenitors such as fibroblasts, endothelial cells, bone marrow-derived mesenchymal stem cells (MSCs) into **“**activated fibroblasts**”** (66–68). These **“**activated fibroblasts**”**, are similar to myofibroblasts, express higher levels of αSMA, collagen 11-α1 (COL11A1), PDGF receptor (PDGFR) α/β and lower levels of caveolin-1 acquiring greater contractile activity and ability to actively proliferate (69). Molecular markers to identify CAFs are not yet fully identified, making it difficult to better elucidate the biology of these heterogeneous and complex classes of cells (70). What is well known is that these kinds of cells, once activated, are able to release TGFβ, promoting tumor progression by inducing the generation of a permissive microenvironment (71).

### The role of IL-6

Once stimulated by TGFβ, CAFs are able to release cytokines and chemokines that attract to the site of tumor lesion immune cells such as neutrophils, macrophages, lymphocytes and natural killer (NK) cells, inducing the reparative inflammatory response that promotes cancer progression (68). Among the plethora of TGFβ-induced cytokines, interleukin 6 (IL-6) is the best known to be linked to an increased risk of developing a wide variety of tumor histotypes (72). IL-6, binding its receptor on tumor cells membrane, activates the IL-6-JAK-STAT axes and the NOTCH signaling pathway promoting cell proliferation and the acquisition of an invasive phenotype (73). Moreover, when synthesized by tumor cells, IL-6 acts in a paracrine way inducing fibroblast differentiation into CAFs. Thus, IL-6 represents a central hub in the close tumor-stroma dialogue necessary for tumor growth and progression (74).

### The role of TNFα

During early stages of tumorigenesis another cytokine is secreted and released into the TME: TNFα that has an established role in angiogenesis, chronic inflammation, tissue remodeling, tumor growth. and in metastases (75). Also in this case, TNFα could exert either tumor inhibition or tumor promotion, depending on the cellular context and cancer stage. Studies revealed that, although TNFα inhibit cell proliferation (76), tumor cells as in a B16 murine melanoma model, secrete low levels of this cytokine promoting infiltrating myeloid cells recruitment, vascularization and progression of the cancer (77). Moreover, in a murine xenograft model of ovarian cancer, expression of TNFα receptor 1 (TNFR1) on CD4+ T cells surface is necessary for stimulation of IL-17 secretion and consequent recruitment of myeloid cells into tumors, thus supporting inflammation and cancer progression (78).

### ECM remodeling and inflammation: a vicious cycle

Beyond mediating the tumor and surrounding stroma interaction, CAFs also secrete molecules belonging to the ECM, a physiological process dysregulated in the early stages of cancer (79–81). This latter phenomenon induces pathological changes in the abundance, structure and architecture of the ECM, influencing tumor growth and progression (82). The deposition of collagen secreted by CAFs, and the changes in the expression of remodeling enzymes, induces an alteration of the collagen fibers organization resulting in an increased ECM stiffness (83). For example, it has been reported that in breast cancer, collagen fibers become linearized and oriented perpendicular to the tumor boundary, thus promoting tumor cell invasion and migration (84). Moreover, in some tumors the cross-linking of collagen and elastin fibers mediated by LOX is increased. In particular, LOX expression is upregulated in many tumors, for example mesothelioma, downstream of hypoxia (HIFs) by a mechanism that is, in part, driven by TGFβ (22, 85, 86). An unbalanced increase in LOX expression and activity promotes an increase in ECM stiffness that induces mechanical activation of latent TGFβ and fuels the initiation of a vicious cycle that maintains an inflammatory environment and promotes tumor progression (83). The activity of LOX enzymes is therefore not limited to shaping the architecture and mechanical functions of the ECM, but is part of a complex bidirectional signaling network within the cellular matrix and microenvironment that helps to establish and maintain an inflammatory, immunosuppressive microenvironment and a pro-tumorigenic TME (22). The close parallelism between fibrosis and desmoplastic matrix involves not only CAFs (called myofibroblasts in fibrosis) but also M2 polarized macrophages and the hyper-reactivation of the TGFβ signal and LOXs and the related epithelial to mesenchymal transition (EMT) a functional transition of polarized epithelial cells into mobile and ECM component–secreting mesenchymal cells. Furthermore, TGFβ is in close cross talk with the desmoplastic matrix in the induction of EMT (87). The activity of proteases that degrade the ECM, for example, induces the release of matrix-bound growth factors and cytokines, which can mediate the activation of downstream effectors, such as oncogenic transcription factors as NF-kB (63, 79), the main inflammatory mediator involved in tumorigenesis. Transient activation of NF-κB is a high regulated molecular process that, under physiological conditions, promotes inflammation as an adaptive and physiological response. However, in cancer, NF-κB is constitutively activated by proinflammatory cytokines, mutated oncogenes and anti-oncogenes via both autocrine and paracrine pathways (88). This persistent activation leads to transcriptional regulation of different ECM components and ECM-remodeling enzymes, such as MMPs, whose expression is under the control of NF-κB (22). MMPs induced by pro-inflammatory factors and regulated by NF-κB (89), have an important role in supporting all the major characteristics of cancer (90). Furthermore, there is a close relationship between cytokine release and specific MMP enzymes that degrade the ECM, since MMPs may play a non-proteolytic and non- ECM role in cell-cell communication (91).

### The effects of chronic inflammation on the TME

Chronic inflammation is considered a common feature in the development and progression of cancer (92). In particular, breast cancer patients often exhibit chronic low-grade inflammation, and the disease bad prognosis is often associated with fat accumulation and fat inflammation (93). Concomitantly, growing evidence show that the inflammation of adipose tissue is a key driver of estrogen production and other pro-inflammatory factors in obese postmenopausal women, which plays an important role in ER+ breast cancer (94). Moreover, chronic inflammation is responsible for the excessive and harmful ROS production with the consequent decaying surrounding adipose tissue to form cancer associated adipocytes (CAAs). With the release of pro-inflammatory adipokines, such as leptin, activating transcription factors, such as NF-κB, and various inflammatory mediators, CAAs are activated and large quantities of chemokines and prostaglandins are produced (60). Various inflammatory cells such as tumor associated macrophages (TAMs), myeloid-derived suppressor cells, neutrophils and mast cells are recruited and further promoted. This process, by influencing cell proliferation and survival, promoting angiogenesis, inhibiting the antitumor immune response, favoring tumor cell infiltration and metastasis, finally, mediates the onset and development of tumors (95). Persisting chronic low-grade inflammation induces in the TME the malignant transformation of TAMs. In particular, upon STAT3 activation, macrophages in the stroma of tumors tend to differentiate into TAMs with M2 characteristics. This kind of macrophage polarization induces a series of events such as the inhibition of the high state of lactic acid produced by glycolysis, the reduced migratory capacity of mononuclear macrophages and the reduced release of TNFα and IL-6. Last but not least, M2 polarization inhibits its antigen presentation function, thus favoring the onset and persistence of immune escape (96).

## Tumor state transition driven by microenvironment forces

The neoplastic transformation of cells and tumor progression are not solely reliant on the accumulation of mutations in oncogenes or tumor suppressor genes, but also on the alteration of cellular communication and interaction with the surrounding environment (97, 98). Quiescent cell clones with mutations in oncogenes or tumor suppressors, which are found in healthy tissues, are incapable of initiating tumor growth (99, 100). While constitutive activation of oncogenic pathways is insufficient to reprogram and promote neoplastic transformation of breast and pancreatic cells in a soft microenvironment that resembles that of healthy tissue, a rigid microenvironment seem to favor tumor formation (101). Therefore, oncogenic reprogramming of normal cells is dependent on both cell-autonomous events, such as oncogenic activation, and non-cell-autonomous events, such as increased stiffness of the environment. Oncogenic signaling enhances cellular mechanotransduction in response to physical inputs received from the environment. Subsequently, even a relatively modest increase in substrate rigidity of the microenvironment, in which oncogene-expressing cells are embedded, is sufficient to ignite oncogenic mechano-signaling and empower oncogene-mediated cell reprogramming (101). Only cells expressing oncogenes respond to sub-threshold mechanical stimuli for healthy cells by increasing contractile actomyosin bundles, heightened by elevated phospho-myosin light chain (pMLC), enhancing the formation and maturation of focal adhesions (FA), and YAP/TAZ activation, as evidenced by their nuclear accumulation (102).

Imbalances in ECM deposition and degradation lead to the accumulation of matrix proteins in the TME (7). Covalent interfiber bonds enable the ECM to withstand external loads, leading to a highly elastic behavior. The cycles of proteolytic degradation, new synthesis, and crosslinking enable permanent remodeling of elastic matrices, resulting in permanent alignment of collagen fibers perpendicular to the tumor margin, which is linked to worse cancer outcomes (103). An increased elastic modulus enhances the ECM**’**s resistance to the force generated by tumor and CAFs via ROCK-dependent actomyosin contractility, contributing to increased cell and ECM stiffness (104). These contractile forces lead to increased hydrostatic pressure and tensile stress on F-actin fibers, which are transferred to the ECM through integrin interaction.

Deregulated cell growth contributes to tumor stiffening, which leads to increased cell density and ECM elastic modulus (105). The basal membrane (BM), which acts as a physical barrier, restricts tumor growth within a limited space (106). The tension exerted by the expanding tumor affects tissue shape and can stimulate the proliferation of neighboring healthy cells. Additionally, tumor pressure can be increased by interstitial fluid pressure due to absorption of water into extracellular proteins from hyper permeable tumor vessels and a disrupted lymphatic system (107). The pressure exerted by the interstitial fluid, BM, and healthy tissue, tumor proliferation and elastic ECM slow down the cellular motion, causing cells to acquire a spherical shape and transition from an active fluid-like state (known as unjamming) to a passive solid-like state (known as jamming) (108, 109).

The considerable compressive stress to which the tumor interior reaches results in cell cycle arrest, whereas tensile stress at the tumor periphery can lead to heterogeneous proliferation rates across the tumor (110). In this context tumor cells trapped in solid like state require to transit from jammed to unjammed fluid like phase in order to become malignant and proliferate, migrate and disseminate. Ductal carcinoma *in situ* (DCIS) typically grow at high cell density within the confinement of the mammary duct lumina and are indolent, quasi benign lesions as they enter a solid (jammed) and kinetically arrested state (111, 112). However, external stress can force some cancer cells to undergo a solid-to-liquid, or jammed-unjammed, phase transition. This change in cellular behavior is induced by the modulation of genes such as RAB5, E-cadherin, and p120 catenin, which promote this phase transition by regulating cell-cell adhesion, substrate adhesion, cell activity (such as traction generation or contractile stress), cell stiffness, cell polarity, cytoskeletal deformability, proliferation, and tumor cell shape (113, 114). In the unjammed state, cells acquire an elongated morphology, which promotes tissue fluidity. This particular cell shape allows for increased mobility by increasing the number of degrees of freedom, reducing geometric constraints, and necessitating the coordination of multiple neighboring cells to restrict its movement (115).

Although the factors that drive unjamming in different contexts are not yet clear, including the role of inducing signals, transcriptional regulators, and downstream effectors (111, 116–118), it can occur independently EMT. While epithelial cells can undergo to unjammed transition in the absence of EMT and move collectively without losing their barrier function, it remains uncertain whether EMT can occur without unjamming (119).

In dense tumors, cancer cells display various dynamic behaviors, where unjammed cells become trapped within a globally jammed layer (120). This encasement of fluid cells by jammed cells results in increased tension network and tissue rigidity (120). Cells and epithelial sheets can adapt to acute stress by activating a nuclear mechanoprotective response that protects them from extensive genomic damage. This response involves increased nuclear rigidity and size, heightened chromatin compaction, and the reorganization of peri-nuclear cytoskeletal actin to form actin rings (114, 121–123). The cell**’**s mechanical constraints, resulting from the surrounding microenvironment, are transmitted to the nuclear envelope via the cytoskeleton network or compressive stress. This can cause nuclear envelope rupture, allowing an exchange of transcription factor and nuclear contents between the cytoplasm and nucleus. The extrusion of DNA from the nucleus generates shear stress, which leads to an increase in γH2AX foci, a sign of DNA damage and double-strand breaks. These breaks can result in significant genomic alterations and activation of cGAS-mediated inflammatory (92, 114, 121, 124–126). Pleomorphisms, or the variance in nuclear shapes and sizes, are crucial clinical markers for tumor aggressiveness and correlate with the tumor cell unjamming transition (127).

Increased tumor stiffness can promote the assembly of focal adhesions, which in turn leads to EMT. Focal adhesions are known to recruit various protein kinases and adaptor molecules, such as p130Cas and paxillin, and function as signaling hubs by activating Src-family kinases, FAK, RHO-family GTPases, and ERK, while also inhibiting the tumor suppressor PTEN (128, 129). As a result, rigid substrates promote the activation of transcription regulators YAP1 and TAZ, which in turn promotes the expression of pro-proliferative and pro-migratory genes (130). Additionally, stiff substrates reduce the association of TWIST1 with G3BP2, promoting the activation of EMT (131). Adhesion to stiff surfaces affects the balance of F-actin and G-actin levels. Reduced G-actin levels release MKL1 transcriptional regulators, leading to increased expression of genes promoting proliferation and migration (132). The expression of ECM proteins, such as CTGF and CYR61, as well as pro-contractile cytoskeleton components induced by YAP1 and SRF activation, forms a positive feedback loop that reinforces matrix stiffness (130, 133).

EMT results in loss of cell-cell adhesion, enhanced mobility, and reduced tissue integrity, affecting cancer cell stiffness (134). When tumor cells move through tissue, their nuclei undergo significant deformation and communicate with the actomyosin cortex via stretch-sensitive proteins in the nuclear envelope (124, 135). *In vitro*, nuclei passing through narrow constrictions can result in nuclear envelope rupture, DNA translocation, and the activation of inflammatory responses (92, 136). Although these ruptures are usually repaired quickly, they can still cause double-strand breaks in DNA, which can contribute to mutagenesis and genetic instability in invasive cells (137). DNA damage and nuclear envelope rupture markers are frequently observed in the invasive margins of both mouse and human mammary tumors (138). Moreover, compression of the nucleus can result in epigenetic consequences, including the formation of heterochromatin in cancer cell lines, which is associated with increased invasiveness.

EMT and unjamming state transition lead to a reduction in cell-cell adhesion and an increase in motility, which ultimately impairs tissue integrity. This phenomenon facilitates tumor growth and cancer cell invasion, resulting in decreased cancer cell stiffness (139).

The tumor development process is a multifaceted phenomenon influenced by genetic elements and the chemical-physical properties of the microenvironment. This interaction triggers mechanosensing pathways, which cause the remodeling of the physical and phenotypic properties of tumor cells. As a result, tumor growth repression mechanisms are bypassed, enabling the invasion of tumor cells from dense tissues into more relaxed distant tissues.

## Signaling pathways in mechanotransduction as potential targeted therapies

**Figure 1:**
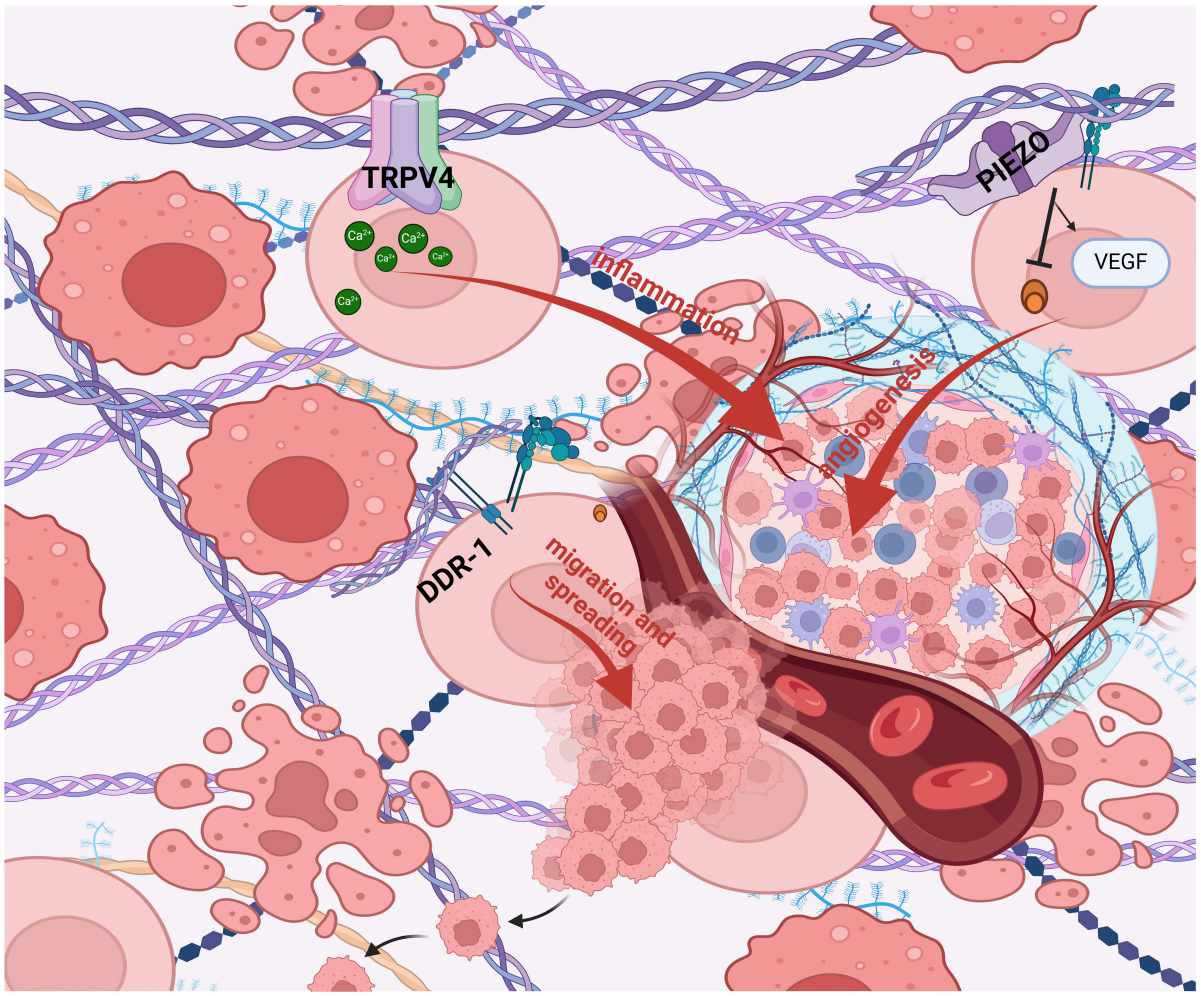
Mechanotransduction in tumor progression. TME, mechanical forces and stiffness of the ECM activate cell- membrane receptors (i.e. DDR1) and ion channels (i.e. PIEZO1; TRPV4)-induced intracellular signaling pathways involved in the cancer hallmarks such as inflammation, angiogenesis, and migration and spreading (140). (BioRender.com).

Hypothesizing that targeting mechanotransduction players could have an anti-tumor effect by influencing the cross talk between stromal cells and the matrix, and given the importance and key role of cellular mechanotransduction in tumor progression, several molecular targets and related drugs have been identified and many of them show anticancer properties in preclinical studies. In particular, TME, mechanical forces and stiffness of the ECM activate cell-membrane receptors (i.e. DDR1) and ion channels (i.e. PIEZO1; TRPV4)-induced intracellular signaling pathways involved in the cancer hallmarks such as inflammation, angiogenesis, and migration and spreading (140) (**Figure 1**). The research on potential mechanotherapy focused on collagen as the main actor in the formation of the stiff matrix. On this issue, several intracellular signaling pathways involved in the synthesis, folding and secretion of collagen were targeted, such as Hsp47 and TGFβ. Small molecules (141) and SiRNA containing nanoparticles targeting these two molecules inhibited hepatic, pancreatic and lung fibrosis (142, 143). Hyaluronic acid has been considered a prognostic biomarker associated with poor prognosis in patients with various types of cancer, such as pancreatic, breast, and ovarian cancer (144–146). Changes in hyaluronic acid metabolism, content and deposition in inflammation and cancer are generally related to the modulation of hyaluronic acid synthesizing (HAS) and degrading (HYALS) enzymes (90). Inhibition of HAS2 activity can be accomplished by drugs such as 4-methylumbelliferone (4-MU) (147, 148). In particular, 4-MU is a chemo-preventive and therapeutic agent used to treat prostate cancer (149). Additionally, treatment of estrogen ER+ breast cancer cells with 4-MU, was shown to reduce cell migration, adhesion and invasion (150). Moreover, the use of HA-degrading hyaluronidase (HYAL) in breast cancer therapy has been approved by the FDA, demonstrating that hyaluronic acid plays a crucial function in cancer progression (151). Other potential mechanotherapeutic targets are represented by the LOX family members (152) and the Hippo Pathway, that regulates the YAP-TAZ nuclear transcription, correlates with EMT, malignancy therapy resistance and metastasis (153). In particular, LOX and the other members of its LOXL family deaminate lysine and hydroxylysine residues by promoting collagen and elastin crosslinking and consequently extracellular matrix stiffening. Therefore, inhibition of LOX and LOXL with specific molecules such as beta-aminopropionitrile (BAPN), which irreversibly binds to the catalytic domain, or with antibodies directed to the catalytic domain of LOXL2, such as simtuzumab, or by inactivation of bone morphogenetic protein 1 (BMP1), which activates LOX by cleavage, or the use of copper chelators, such as tetrathiomolybdate, which is essential for the activity of the catalytic domain of LOX, has been shown in preclinical studies to improve the anti-tumor response of therapies in several tumor types. The efficacy of such compounds, such as simtuzumab, antibody AB0023, Tetrathiomolybdate, in combination with different therapies, has been tested in several phase I-III clinical trials in cancer patients (154–160) (**Table 1**). Another strategy to prevent extracellular matrix stiffness involves the use of antifibrotics that repress collagen synthesis and maturation by inhibiting SMAD3 with halofuginone or TGFβ with pirfenidon or losartan in cancer-associated fibroblasts, the major producers of collagen. Several clinical trials are still underway to determine whether the administration of such inhibitors of collagen synthesis can improve the prognosis of patients undergoing various cancer therapies (161–166) (**Table 1**). Collagen degradation can be induced by the incorporation of proline analogs, such as thiaproline, which destabilize the triple helix structure of collagen. This approach has been found to be effective in preclinical mesothelioma models. In the tumor microenvironment, CAFs are the major producers of collagen; therefore, depletion of these cells using CAR-Ts specific for surface proteins, such as fibroblast activated protein (FAP), mainly expressed by CAFs, is another promising strategy for reshaping the tumor microenvironment and making it more accessible to drugs and immune cells. Clinical trials are currently evaluating the efficacy of CAR-T directed against FAP in lung tumors (167–170) (**Table 1**). Alignment of collagen fibers directed by discoidin domain receptor 1 (DDR1) makes the microenvironment less accessible to immune system cells and consequently generates a pro-tumoral environment, thus preventing immune recognition and response (**Figure 1**). Antibodies directed against DDR, such as PRTH-101, have shown good efficacy in inhibiting the growth of breast tumors by increasing immunoinfiltration in preclinical models and are under evaluation in clinical trials conducted in patients with colon or breast cancer (171, 172) (**Table 1**). Another therapeutic strategy under investigation involves the use of adenoviruses expressing genes such as relaxin, decorin, and MMP-8, which can inhibit collagen synthesis, assembly, and degradation, respectively, or the use of bacteria expressing collagenase (collagen I-degrading Salmonella). In preclinical models, the administration of these therapies has shown positive and encouraging outcomes in slowing the growth of pancreatic and renal cancers (173–176). Mechanical and biochemical remodeling of the matrix and changes in tissue stiffness activate, among others, the mechanosensors PIEZO1 and TRPV4 (180). Piezoelectric channels are non-selective, Ca2+-permeable channels that constitute pores activated in response to mechanical stimuli applied to the cell membrane, converting various mechanical forces into biochemical signals (181–183). Deregulation and aberrant expression of *PIEZO1*and *PIEZO2* have been linked to increased tumor proliferation and metastasis and to the mechanotransduction of matrix stiffness (184, 185). Several studies were conducted in order to find selective and promising PIEZO inhibitors, but results on tumor growth and metastasis are still unsatisfactory (186). The high expression of TRPV4 is characteristic of epithelial tissues and of the tumor counterpart, these receptors responding to heat, osmotic changes and mechanical stretching, inducing the influx of Ca2+, TRPV4 also regulates cell volume by interacting with F-actin (187–190). TRPV4 also promotes the process of EMT caused by matrix rigidity, upstream of metastatic dissemination, enhancing activation of the Akt kinase and the translocation of YAP/TAZ into the nucleus (191). Small molecules and monoclonal antibodies aimed at TRPV4 inhibition are in early phase clinical trials (192, 193).

**Table 1:** Mechanotransduction's molecular target and related drugs.

Drug	Target	Histology	Current stage	Clinicaltrials.gov identifier (NCT number)	References
Beta-aminopropionitrile (BAPN)	LOX inhibitor	Pancreas, breast and colon cancer	Precinical	–	(154)
Simtuzumab	LOX inhibitor	Pancreas, breast and colon cancer	Phase II	NCT01472198 NCT01479465	(155)
PXS compounds	LOX inhibitor	Breast, oral cancer and hepatocellular carcinoma	Phase I	NCT05109052	(156, 157)
antibody AB0023	LOX inhibitor	Breast and melanoma cancer	Precinical	–	(158)
Tetrathiomolybdate (TM), a copper chelator	LOX inhibitor	Breast, prostate, melanoma, hepatocellular, esophageal, and head neck squamous cell carcinoma cancer	Phase II Phase I Phase II Phase I Phase II	NCT00150995 NCT01837329 NCT00195091 NCT00006332 NCT00176800	(159, 160)
Halofuginone	Smad3 inhibitor	Pancreatic, Hepatocellular carcinoma, Sarcoma	Phase I Phase II	NCT00027677 NCT00064142	(161, 162)
Pirfenidone	TGFβ inhibitor	Non- small cell lung cancer, breast cancer	Phase I/II	NCT04467723 NCT05704166	(163, 164)
Losartan	TGFβ inhibitor	Sarcoma, melanoma, and pancreatic breast cancer	Phase I/II Phase II Phase II Phase I	NCT00880386 NCT05637216 NCT05077800 NCT03864042	(165, 166)
Thiaproline	collagen triple-helix conformation	Mesothelioma	Preclinical	–	(167)
anti-FAP CAR-T	CAF	Lung Cancer	Phase I	NCT01722149	(168–170)
PRTH-101 (DDR1-neutralizing antibody)	collagen receptor discoidin domain receptor 1 (DDR1)	Breast and colon carcinoma	Phase I	NCT05753722	(171, 172)
Oncolytic adenoviral viruses	Relaxin, decorin and MMP expression	Pancreatic, renal cell carcinoma	Preclinical	–	(173–176)
Engineered collagen I-degrading Salmonella typhimurium	Collagen-I degradation	Pancreatic cancer	Preclinical	–	(177)
TRPV4 inhibitor	TRPV4	Colorectal, lung, and gastric cancer	Preclinical	–	(178, 179)
Piezo targeted drugs	Piezo 1	Breast, gastric, bladder, lung, prostate, colon cancer	Precinical	–	(180)

## Discussion

Solid tumors are characterized by a rigid matrix and altered ECM components which translates into an increase in interstitial fluid pressure and chemo resistance given by the lack of perfusion of stiff tumor tissues to drugs (194). These evaluations push several studies towards strategies that, by targeting the components of the ECM, develop therapeutic agents that favor the perfusion and release of drugs within tumor cells and stroma, capable of extending the current strategies of precision medicine by expanding the range of targets also to the constituents of the altered matrix. Therefore, while genetic modifications in tumor cells undoubtedly initiate and guide the malignancy of the tumor in an incisive way, favoring neoplastic progression, the tumor stroma and its matrix play, with each other, a fundamental and synergistic role. Indeed, since the tumor progresses within an ECM, the matrisome is in its dynamic evolution and in close cross-talk with the oncosome. We can therefore hypothesize a “Matrix Addiction” process with one or several matrisome drivers, with a prevalent function in some contexts and tumor histotypes, to be combined in synergistic way with the genes driving the “Oncogene Addiction” process (195). This fine and yet not fully explored dynamic balance modulates virtually every tumor hallmark, reciprocally influencing cancer-associated stromal cells, responsible for the most part of the matrix remodeling, creating a feedback loop between ECM, stromal cells and tumor cells, in a continuously evolving mutual dynamic manner. Changes in qualitative and quantitative variations in composition, architecture and cell-ECM interactions have impactful consequences on cellular and tissue functions. These processes are regulated on multiple levels to preserve tissue homeostasis and involve the interaction of different cell types. Alterations in ECM remodeling play a critical role in cancer development and progression. The collagen IV protein is critical for the maintenance of BM structure and function. Laminin is sufficient to maintain the structure of the BM in the early stages, although collagen IV is responsible for maintaining membrane stability and integrity. Therefore, abnormal expression of laminin and collagen is a hallmark of some cancers. The BM was previously thought to be invaded by protease degradation, but with experimental progress, it can be observed that cancer cells also have physical factors that facilitate this invasion. Several immune cells contribute significantly to changes in the ECM and are also functionally affected by these changes. It should also be considered that advanced proteomic analysis provides adequate tools to study the entire tissue matrisome in tumor and non-tumor pathological contexts. An interesting research direction centers on new 3D technologies that shed light on the cellular context of ECM remodeling and cell-ECM interactions at single and subcellular levels. With these new advanced technologies, biomedical research can adequately approach and understand the global interactions of individual cells in their environment, which could lead to the development of more effective ECM-targeted therapies involving more realistic precision therapy because considers not only the mutational alterations and PTM of the oncosome but also those of the matrisome. The ultimate aim of this review is to underline how the complexity of the redundant interactions between the matrisome and the oncosome and their variants PTM, Driver mutations, alternative splicing) translate into the clinical reality of personalized therapy, forcing us to evaluate and target both compartments in synchrony (196–198).

## Author contributions

AM: Funding acquisition, Writing – original draft, Writing – review & editing. MTG: Funding acquisition, Writing – original draft, Writing – review & editing. FP: Writing – original draft, Writing – review & editing. SC: Writing – review & editing, Writing – original draft. MG: Supervision, Writing – review & editing. AG: Supervision, Writing – review & editing.
